# Effect of educational intervention based on PRECEDE model on lifestyle modification, self-management behaviors, and hypertension in diabetic patients

**DOI:** 10.1186/s12902-023-01264-y

**Published:** 2023-01-06

**Authors:** Ali Khani Jeihooni, Ali Sobhani, Pooyan Afzali Harsini, Mehdi Amirkhani

**Affiliations:** 1 Nutrition Research Center, Department of Public Health, School of Health, Shiraz University of Medical Sciences, Shiraz, Iran; 2 Department of Public Health, School of Health, Fasa University of Medical Sciences, Fasa, Iran; 3 Department of Public Health, School of Health, Kermanshah University of Medical Sciences, Kermanshah, Iran; 4 Department of Nursing, School of Nursing, Fasa University of Medical Sciences, Fasa, Iran

**Keywords:** Life Style, Diabetes self-management, Diabetes Mellitus, Hypertension, PRECEDE model

## Abstract

**Background:**

Inappropriate lifestyle and poor self-management in diabetic patients lead to many complications including hypertension and increased disease burden. Because of insufficient studies on Effect of educational interventions on lifestyle, self-management and hypertension in diabetic patients, the present study aimed to evaluate the Effect of educational intervention based on PRECEDE model on lifestyle, self-management, and hypertension of diabetic patients.

**Methods:**

This clinical trial was conducted on 300 diabetic patients with hypertension. The patients were selected using simple random sampling and divided into 2 groups of intervention (150 people) and control (150 people). The intervention group was trained through ten 50–55 min sessions on lifestyle skills, self-management, and hypertension control based on the PRECEDE model. Before and after the intervention, lifestyle skills, self-management, and PRECED model constructs were evaluated using a standard questionnaire. Data were analyzed by SPSS 20 software using t-test, Kolmogorov–Smirnov, and Chi-Square tests (*P* < 0.05).

**Results:**

In the intervention group, the mean score of different dimensions of lifestyle and self-management significantly increased from 110.45 ± 18.78 to 172.58 ± 186.66 and 64.33 ± 15.24 to 144.32 ± 15.82, respectively (*P* = 0.001). Mean systolic and diastolic blood pressure also decreased from 148.5 ± 5.39 to 123.54 ± 5.32 and 95.41 ± 3.12 to 72.24 ± 3.06 (*P* < 0.001). Moreover, the mean score of all the PRECEDE model constructs significantly increased after the intervention. In the control group, the mean score of the PRECEDE model constructs, the dimensions of lifestyle, self-management, and systolic and diastolic blood pressure did not change significantly before and after the intervention (*P* > 0.05).

**Conclusion:**

‌ Based on the study's results, the PRECEDE model was found to be a non-invasive, non-pharmacological, cost-effective method without any complication and as a complementary action along with other methods in the treatment of diabetic patients.

## Background

Chronic diseases are one of the main issues affecting human health as life expectancy rises [1]. One of the most common chronic illnesses, diabetes is referred to by the World Health Organization as a hidden pandemic (WHO). According to previous study, if persons with diabetes effectively manage their hypertension, many complications may be prevented. [2]. Increasing patients' physical and mental function through lifestyle changes is the most important part of treating chronic diseases [3]. When a person is aware of the positive Effect a healthy lifestyle has on an illness and its complications, they are more likely to engage in healthy habits [4]. Diabetics' capacity to manage their hypertension can be significantly impacted by educational interventions and the adoption of morally sound lifestyle and self-management practices [5]. Self-management is the cornerstone of diabetes treatment, and providing patients with diabetes with self-management education is essential [6]. Self-management is an active and practical process directed by the patient [7]. If diabetic patients are to successfully manage their condition, lead a healthy lifestyle, and avoid issues like hypertension, they must have access to enough information [8].

Today, education is one of the most important elements of health care [9]. Healthcare education and promotion emphasize improving lifestyle and self-management in response to people's increasing involvement in health-related activities. Additionally, it is one of the best ways for people to maintain their health, achieved through effective and efficient instructional techniques [10]. Theories and models provide a methodical view of events and are a common way to evaluate successes and failures. They provide information necessary for planning, carrying out, and assessing educational interventions by acting as a map of the educational process [11]. Despite many models for health promotion, studies have shown that the PRECEDE model is more appropriate for designing health promotion programs [12]. This model it consists of the initials of the following words (PRECEDE: Predisposing, Reinforcing, Enabling Constructs in Educational, Diagnosis, and Evaluation) and.

It was designed by Lawrence Green and Marshall Crotter in 1980 to change behavior and examine an educational program's possible outcomes. The model addresses all the educational needs for health promotion from different aspects of values, beliefs, and attitudes and is flexible, acceptable, measurable, and process-oriented [13]. Additionally, it offers a structure for figuring out the enabling, reinforcing, and predisposing elements that affect behavior in educational diagnostics. The most useful use of the model is to explain behavioral aspects [14].

A study by Aghamalai et al. [15] showed that The educational program based on the preceding model could effectively improve a healthy lifestyle in patients with hypertension. Azar et al. [16] showed that an educational program based on the preceding model effectively improves the life quality of hypertensive patients.

The results of a study entitled Effect of educational intervention based on precede model combined with self-management theory on self-care behaviours in type 2 diabetic patients showed that the educational intervention based on precede model alone or combined with self-management theory could effectively improve predisposing, enabling, and reinforcing factors in type 2 diabetic patients [16]. A study by Barasheh et al. [17], Entitled effect of educational program based on the preceding model on improving self-care behaviors in a semi-urban population with type 2 diabetes showed that Precede model would be an appropriate framework to educate patients with type 2 diabetes as well as promote self-care behaviors. The findings of the study of KhaniJeihooni [12] revealed that the design and performance of educational programs based on precede model have an influence on the changes of predisposing factors, reinforcing and enabling factors of overweight students, and caused the reduction of their weight.

Poor lifestyle choices, inappropriate self-management, and the increase of comorbidities such as hypertension persist despite several studies on diabetes education.This study aims to assess the impact of an educational intervention based on the PRECEDE model on diabetes patients' lifestyle, self-management, and hypertension. Furthermore, create a framework that predisposing factors (knowledge, attitude, and perceptions), reinforcing factors (influence of others, family, and peers), and enabling factors (availability of resources and skills) are considered as factors affecting behavior in educational diagnosis [18].

Similar studies have been conducted in this field, but the present study has been done on a larger sample size than similar intervention studies. Also, due to the Covid pandemic, it has used training methods in cyberspace and peer training. On the other hand, the use of educational methods such as group discussion, role-playing, and preparation and distribution of educational videos have slightly differentiated this study from similar studies. The results of similar studies also show that more studies are needed to optimally judge and evaluate the intervention's impact.

## Methods

This intervention study was conducted on 300 type two diabetes mellitus patients with hypertension under the auspices of Fasa Diabetes Center. Based on previous studies [19, 20].

The Fasa Diabetes Center received referrals for patients with type 2 diabetes mellitus and hypertension who matched the enrollment requirements. The inclusion criteria included active cases at the Diabetes Center, a history of diabetes lasting more than ten years, hypertension, no previous medication history (except for anti-diabetic and anti-hypertensive drugs), and underlying illnesses (cardiovascular, neuromuscular, cancer, etc.). Reluctance to participate and absence from more than two sessions were two exclusion criteria.

400 participants with type 2 diabetes mellitus and hypertension were invited to the trial based on the inclusion criteria. Some, though, objected. The remaining 350 patients were split into intervention and control groups randomly, totaling 300 patients (150 patients each).

The data collection instruments included demographic questions, PRECEDE model constructs, a lifestyle questionnaire, and an instrument for diabetes self-management. Age, gender, marital status, monthly household income, education, duration of diabetes, and family history of diabetes were among the demographic variables asked. The following questions evaluated the PRECEDE model's constructs:

Twenty multiple-choice questions (right answer = 1, incorrect answer = 0) were used to assess knowledge. Finally, a score ranging from 0 to 20 was determined for each patient. Ten questions measuring patients' agreement or disagreement with statements were used to assess their attitudes. The responses were based on a 5-point Likert scale ranging from "totally disagree" (score 1) to "absolutely agree" (score 5); hence, each patient received a score between 10 and 50.

The self-efficacy was assessed using ten questions. The answers ranged from extremely high (scoring 4) to extremely low (scoring 0), resulting in a score between 0 and 40 for each patient. Using 10 questions, the enabling variables were evaluated. The responses were based on a 5-point Likert scale ranging from "totally disagree" (score 0) to "absolutely agree" (score 4); thus, each patient received a score between 0 and 40. Ten questions on the support of family members (parents, siblings, etc.), other relatives, friends, doctors, diabetic center personnel, and health center staff were used to assess predisposing factors. The responses were based on a 5-point Likert scale ranging from "totally disagree" (score 0) to "absolutely agree" (score 4); thus, each patient received a score between 0 and 40.

The validity of the items was assessed by generating an item effect score of 0.15 or higher and a content validity ratio of 0.79 or higher. To assess the face validity of the measure, forty hypertensive diabetics with comparable demographic, economic, and social characteristics were asked to choose options from a list. The opinions of 12 professionals in health education and health promotion, including one nurse and one nutritionist, were used to determine the content validity. Lawshe index values over 0.56 were considered critical, and those items were kept for research. Most goods have a 0.70 or above. Cronbach's alpha was used to get the dependability score, which came out to be 0.89. The computed reliability for knowledge, attitude, self-efficacy, predisposing factors, and enabling factors, respectively, was 0.86, 0.88, 0.84, 0.82, and 0.89.

The questionnaire contained 52 questions that evaluated health-promoting habits across six dimensions: nutrition, physical activity, health responsibility, stress management, interpersonal relationships, and self-actualization. The responses varied from never (score 1), occasionally (score 2), frequently (score 3), and always (score 4). (score 4). 2011 saw the Iranian version of the questionnaire validation by Mohammadi Zaidi et al. The content validity and reliability of the questionnaire were determined to be 82% [21].

The questionnaire was created by Walker and Pender in 2008 and had 35 questions over five dimensions. Self-management includes self-integration, self-regulation, engagement with medical experts, self-monitoring, and adherence to the prescribed treatment plan. Self-integration (10 questions) is concerned with the ability of people with diabetes to combine daily activities with diabetes, whereas self-regulation (9 questions) is concerned with the ability of patients to self-regulate their behavior by monitoring the physical symptoms of diabetes. In addition, nine questions assessed interaction with health experts and influential persons, four assessed self-monitoring, and three assessed adherence to the planned treatment regimen. On a 5-point Likert scale, responses ranged from "totally agree" (scoring 1) to "absolutely disagree" (score 5). (score 5). 2011 saw the validation of the Iranian version of the instrument by Tol et al. The questionnaire's content validity was established, and its reliability was evaluated as = 87% [22].

The current investigation was approved by the ethics committees of the Fasa University of Medical Science and the Fasa Diabetes Center. Participants also supplied written consent and received assurances that the information they submitted would be kept private. The control group participated in a 4-h educational session once the study was over.

Prior to the intervention, the surveys were finished by both groups. At the beginning of the procedure, blood pressure was measured, both systolic and diastolic. The educational intervention next included ten 50–55 min lectures, Q&A sessions, group discussions, real-world demonstrations, video clips, and PowerPoint presentations, depending on the outcomes of the pre-test. The sessions covered the definition of hypertension, issues with uncontrolled hypertension, healthy eating, regular medication, ongoing hypertension control, behavioral change (such as quitting smoking and drinking alcohol and managing stress), self-efficacy, interpersonal support, and responsibility. The role of storytellers was discussed in one of the meetings that a family member, staff members from the Diabetes Center, and the doctor attended.

Finally, a WhatsApp group was established, and the patients received a brochure. The WhatsApp group received motivational and instructive messages every day. Participants in the training phase had the opportunity to interact with one another, share knowledge, and ask questions to advance their experience and skills.

Patients were divided into groups of 15 to 20 and provided information at various periods to improve outcomes and emphasize the value of friends and support networks. Every two weeks, participants in the control and intervention groups were asked to take a blood pressure reading to motivate them to stay in the study. Blood sugar checks were done monthly in addition to hypertension control to encourage patients to continue their studies. After three months, the mean hypertension of six hypertension controls was calculated. Using a single calibrated pressure gauge, the researchers themselves evaluated hypertension. Both groups answered questions about the intervention three months later. No samples were lost while the study was underway. The data were analyzed using SPSS 22 software, Chi-square, independent t-tests, and t-pair tests.

## Results

In this study, 300 diabetic patients with hypertension were assessed under the supervision of the Fasa Diabetes Center. The intervention and control groups had mean ages of 52.358.20 and 54.108.08 years, respectively (*P* = 0.263). The mean duration of diabetes in the intervention and control groups was not significantly different (*P* = 0.304): 19.185.22 and 18.875.10 years, respectively.

According to the Chi-square test, there was no statistically significant difference between the two groups in terms of education (*P* = 0.190), monthly household income (*P* = 0.289), family history of diabetes (*P* = 0.314), married status (*P* = 202) and gender (*P* = 0.281). (Table 1).

**Table 1:** Demographic information of studied patients

Variables		Intervention group		Control group		*P*-value
		number	percentage	number	percentage	
Education	Illiterate	3	3	2	1.33	0.190
	Primary school	14	14	10	6.67	
	Secondary school	36	36	42	28	
	High school	65	65	68	45.33	
	University	32	32	28	18.67	
Gender	Female	86	86	79	52.67	0.281
	Male	64	64	71	47.33	
Marital status	Single	9	9	6	4	0.202
	Married	129	129	134	89.33	
	Divorced	9	9	6	4	
	Widowed	3	3	4	2.67	
History of diabetes	Yes	Yes	34	30	20	0.314
	No	No	116	120	80	
Household monthly income	< 20,000,000 Rials	< 20,000,000 Rials	42	37	24.67	0.289
	20,000,000-50,000,000 Rials	20,000,000-50,000,000 Rials	73	71	47.33	
	>50,000,000 Rials	>50,000,000 Rials	35	42	28	

Before the educational intervention, independent t-tests revealed no significant differences between the groups in knowledge, attitude, self-efficacy, enabling, and strengthening factors; however, three months after the educational intervention, the intervention group demonstrated a significant increase (Table 2).

**Table 2:** Comparison of mean score of PRECEDE model constructs in the experimental and control groups

variable	group	before intervention M±SD	3 months after the intervention M±SD	Mean difference	*p*-value
knowledge	experimental	7.45±1.89	16.76±1.97	-9.31	0.001
	control	7.70±1.67	8.34±1.66	-0.64	0.284
	*p*-value	0.175	0.001		
attitude	experimental	21.27±4.23	43.14±4.28	-21.87	0.001
	control	21.94±4.11	22.78±4.31	-0.84	0.261
	*p*-value	0.193	0.001		
self-efficacy	experimental	12.16±3.38	34.13±3.58	-21.97	0.001
	control	12.90±3.29	13.81±3.38	-0.91	0.159
	*p*-value	0.317	0.001		
reinforcing factors	experimental	14.10±3.17	33.36±3.44	-19.26	0.001
	control	13.92±3.30	14.64±3.39	-0.72	0.148
	*p*-value	0.322	0.001		
enabling factors	experimental	9.98±2.20	32.14±3.18	-22.16	0.001
	control	11.74±2.23	13.12±2.28	-1.38	0.159
	*p*-value	0.144	0.001		

According to the independent t-test, there was no significant difference between the two groups in terms of lifestyle dimensions (nutrition, physical exercise, responsibility, stress management, interpersonal support, and self-actualization) prior to the educational intervention; however, three months after the intervention, there was a significant difference between the intervention and control groups. In addition, the t-pair test revealed a substantial increase in lifestyle characteristics in the intervention group, whereas there was no significant change in the control group (Table 3).

**Table 3:** Comparison of mean score of lifestyle dimensions in the experimental and control groups

variable	group	before intervention M±SD	3 months after the intervention M±SD	Mean difference	*p*-value
Nutrition	experimental	19.14±2.94	28.54±2.68	-9.4	0.001
	control	18.74±2.78	19.30±2.72	-0.56	0.186
	*p*-value	0.218	0.001		
Physical exercise	experimental	20.22±2.14	27.24±2.17	-7.02	0.001
	control	21.55±2.10	22.17±2.19	-0.62	0.206
	*p*-value	0.185	0.001		
Responsibility	experimental	18.24±3.19	29.10±3.12	-10.86	0.001
	control	19.70±3.07	20.72±3.03	-1.02	0.194
	*p*-value	0.229	0.001		
Stress control	experimental	16.89±2.41	27.34±2.52	-10.45	0.001
	control	17.85±2.40	18.69±2.37	-0.84	0.242
	*p*-value	0.236	0.001		
Interpersonal relationships	experimental	19.37±3.17	30.25±3.12	-10.88	0.001
	control	18.90±3.24	19.33±3.25	-0.43	0.208
	*p*-value	0.180	0.001		
Self-actualization	experimental	18.66±3.69	31.10±3.21	-12.44	0.001
	control	19.72±3.75	21.03±3.54	-1.31	0.164
	*p*-value	0.192	0.001		
Total	experimental	110.45±18.78	172.58±18.66	-62.13	0.001
	control	113.38±18.42	119.74±17.97	-6.36	0163
	*p*-value	0.232	0.001		

Prior to the educational intervention, using an independent t-test, there was no significant difference between the two groups regarding diabetes self-management dimensions (self-integration, self-regulation, interaction with health professionals, self-monitoring, and adherence to the proposed treatment regimen); however, 3 months after the intervention, there was a significant difference between the two groups. In addition, the t-pair test revealed a significant rise in diabetes self-management characteristics in the intervention group but no change in the control group (Table 4).

**Table 4:** Comparison of the mean score of the diabetes self-management dimensions in the two groups before and 3 months after the educational intervention

variable	group	before intervention M±SD	3 months after intervention M±SD	Mean difference	*p*-value
Self-integration	experimental	17.22±4.47	42.16±4.35	-24.94	0.001
	control	18.33±4.52	21.44±4.39	-3.11	0.184
	*p*-value	0.204	0.001		
Self-regulation	experimental	16.36±4.26	37.20±4.20	-20.84	0.001
	control	21.12±4.22	18.17±4.18	2.95	0.175
	*p*-value	0.177	0.001		
Interaction with health professionals	experimental	17.57±4.15	36.60±4.74	-19.03	0.001
	control	18.62±4.11	20.58±4.27	-1.96	0.199
	*p*-value	0.203	0.001		
Self-monitoring	experimental	7.08±1.33	16.27±1.40	-9.19	0.001
	control	7.73±1.30	8.56±1.41	-0.83	0.187
	*p*-value	0.199	0.001		
Adherence to the treatment regimen	experimental	6.14±1.08	12.08±1.14	-5.94	0.001
	control	7.02±1.11	8±1.15	-0.98	0.194
	*p*-value	0.179	0.001		
Total	experimental	64.33±15.24	144.32±15.82	-79.99	0.001
	control	68.88±15.22	79.72±15.45	-10.84	0.114
	*p*-value	0.201	0.001		

Before the intervention, there was no significant difference in mean systolic and diastolic hypertension between the two groups; however, three months after the intervention, there was a significant difference. In addition, the t-test demonstrated a substantial reduction in systolic and diastolic hypertension in the intervention group, whereas no significant change was detected in the control group (Table 5).

**Table 5:** Comparison of the mean score of hypertension in the two groups before and 3 months after the intervention

variable	group	before intervention M±SD	Three months after the intervention M±SD	Three months after the intervention M±SD	*p*-value
Systolic hypertension	experimental	148.67±5.39	123.54±5.32	123.54±5.32	0.001
	control	146.98±5.78	147.04±5.75	147.04±5.75	0.312
	*p*-value	0.143	0.001	0.001	
Diastolic hypertension	experimental	95.41±3.12	72.24±3.06	72.24±3.06	0.001
	control	94.99±3.43	95.25±3.44	95.25±3.44	0.348
	*p*-value	0.168	0.001	0.001	

## Discussion

The predisposing factors in the educational diagnosis phase, including disease knowledge and attitude, were explored since the PRECEDE model served as the theoretical underpinning for the current study. Following the intervention, there were substantial differences between the intervention group and the control group in terms of knowledge, the most significant predisposing factor in the PRECEDE model. a current investigation by Bazrpour et al. [23], there was a significant increase in knowledge immediately and one month after the intervention. The results of our study were consistent with the result of studies by Wang et al. [14], Khani Jeyhooni et al. [24], Koc et al.[25], and Kaewchi et al. [26]. Using group discussion, brainstorming, and participating individuals and peers pave the way to share information and experiences. And this was even more important in our study due to its large sample size. In this study, the educational intervention also had a significant effect on patients' attitudes, which was consistent with studies by Chaboksavar et al. [27], Lin et al. [14], and Hlaing et al. [28]. In actuality, a good education includes both the exploration of values and attitudes as well as the acquisition of knowledge. In the current study, educational techniques, group discussions, and real-world examples, including role-playing, brainstorming, and problem-solving skills, were used to help patients' attitudes. A change in information cannot cause an intended change in action if it does not also cause a change in attitude.

Self-efficacy was another aspect that the educational intervention greatly changed. This study showed that the patients' self-efficacy was low before the study and grew as their knowledge and attitude about the disease improved. However, the management of their disease was considerably impacted by diabetic patients' higher levels of self-efficacy. The results of the present investigation corroborated the findings of Megan et al. research, which were consistent with those results. [29] and Azar et al. [16]. A study by Barasheh et al. [17]. It was demonstrated that interventions leading to enhanced self-efficacy are required to improve the indicators of diabetes and its proper control. After the intervention, this study's mean score of the reinforcing elements increased significantly. Education and support contribute to the development of behavior. In our study, family and friend support had an essential and reinforcing role. Accepting the patient's current status requires family support; support is also essential for chronic patients to manage their condition independently. Our results were consistent with those of other studies because patients' self-care benefits greatly from the support of medical experts, family, and friends. [16, 30, 31]; with the difference in the mentioned studies the sample size and the number of training sessions were less, and also in the study of Solhi et al., they used the self-management theory in addition to the Persed model for educational intervention. The enabling elements significantly changed after the educational intervention due to improvements in knowledge, attitude, self-efficacy, and reinforcing factors. The supporting elements in the current study increased the participants' intent to change their lifestyle and take control of their health, which reduced their blood pressure. Diabetes patients are more motivated to improve their lifestyle and self-management, which reduces complications when they have the necessary knowledge and a positive attitude toward lifestyle modification and self-management, believe they can carry out these behaviors, and receive encouragement from subjective norms like family members, doctors, and diabetes center staff. The educational intervention had a significant positive impact on the enabling factor. Based on Doshmangir et al. [32], We identified attending educational sessions, offering educational resources, and having the capacity to engage in regular physical activity in old age as enabling factors. In line with our findings, the intervention group's mean score of enabling factors dramatically increased after the educational intervention. The difference is that the researcher used the available sampling method in this study, which is less generalizable to other societies. On the other hand, the sample size and the duration of this study were less.

Another important goal of the current experiment was to assess how the intervention affected lifestyle modification. The control and intervention groups did not significantly differ on numerous lifestyle and dietary factors prior to schooling. With an effective educational intervention to increase diabetic patients' knowledge and understanding of nutritional and lifestyle concepts, many complications, including hypertension, can be avoided. The results of a study by Oshwandi and others. [33], The dietary constraints of hemodialysis patients proved that education had little Effect on the nutrition of hemodialysis patients. The findings of this investigation were inconsistent with those of our own. According to Whatnall et al. [29], After receiving the education, the average score for physical exercise in the intervention group increased considerably, showing an increase in patients' understanding and performance of physical activity. The research by Shayesteh et al. [34] also showed that educational intervention increases the knowledge and performance of hypertensive patients in physical activity, which was consistent with our study. A study by Khavoshi et al. [35] also showed that education changes the lifestyle of the elderly regarding physical exercise and nutrition. With the difference in the mentioned study, the increase in the physical activity score after the study was less, probably due to the older population, all older people.

The present study was also interested in personal health responsibility, and based on these findings, the educational intervention increased patients' health responsibility. Ebrahimi et al. [36] showed that educational intervention using mobile phones positively affects educational intervention. In a study by Chafjiri et al. [36], According to our study findings, the lifestyles of 70 elderly patients also improved in the responsibility category. Due to educational interventions, patients have a deeper understanding of their disease and change their behavior toward health obligations due to knowledge and awareness. The findings likewise showed that diabetes patients lacked efficient stress management before the intervention. However, in contrast to Safabakhsh et alfindings,.'s the mean score of stress management dramatically increased after the intervention [37]. The study by Ebrahimi et al. [38], confirmed our findings and suggested that educational intervention delivered via mobile phone has a positive effect on women's sense of responsibility. In order to effectively manage the disease, a diabetic patient's emotional state is essential. In the current study, an improved understanding of the disease's causes and lifestyle changes, along with adjustments to performance and physical activity, resulted in better stress management, which can be highly helpful in treating hypertension. The difference is that the face-to-face teaching method was also used in our study more effectively than in the mentioned study.

Another dimension of lifestyle evaluated in the present study was interpersonal relationships, which significantly increased after the intervention. Compared to other studies, the strengths of our study were special attention to problem-solving skills, brainstorming, critical thinking, and group discussions in improving patients' self-actualization. The intervention group's lifestyle modification increased due to improved diet, physical activity, stress management, health responsibility, interpersonal relationships, and self-actualization, consistent with other studies' findings. (53, 56. 58). lifestyle changes can help diabetics stay healthier and avoid many issues, which can help decrease risk factors, including hypertension.

Aspects of self-management were also regarded in this study as crucial to managing diabetes and avoiding its complications. The results of the study showed no significant difference in the mean self-management score between the two groups prior to the intervention, proving that the samples in both groups were homogeneous. However, education considerably changed the mean self-management aspects score for the intervention group. Self-integration in the intervention group significantly increased after obtaining an education. In the current study, the patient's attitude toward healthcare behaviors was improved by the educational intervention and the patient's increased knowledge and awareness, leading to improved self-management.The educational intervention significantly affected the mean self-regulation score in the intervention group. The results of a study by Habibzadeh et al. [39] showed that diabetes self-management education through group discussion is more effective than usual education programs in the self-regulation dimension. A study by Weng et al. [40] showed a significant effect of educational intervention. This is even though in the mentioned study, only the group discussion method was used, and the educational content was not presented by the lecturer; on the other hand, the number of sessions was limited, and only eight sessions were conducted.

Comparison of the mean score of interaction with health professionals showed the effectiveness of the intervention, which was consistent with the results of studies by Ranaei et al. [19], Habibzadeh et al. [39], and Liu et al. [41]. In contrast to prior studies, our intervention strategy, duration of follow-up, research demographic, and sample size were unique. The mean score for interaction with health professionals was quite low before the intervention but dramatically raised afterward. Improving the patient's engagement with health professionals and workers boosted patient satisfaction and treatment adherence, resulting in a quicker recovery and fewer problems. In many instances, poor interaction results from a lack of communication skills. In the present study, the goal of the sessions was to improve the patient's connection with medical personnel.

After education, the self-monitoring mean score in the intervention group climbed significantly. Self-monitoring is one of the most crucial self-management practices for illness control and complication prevention. In a study by Ranaei et al., the educational intervention had no effect on self-monitoring, which contradicts our findings. Baptista et al. [42] research showed the significant Effect of educational intervention on the self-monitoring of blood glucose. The results of a study by Mayor et al. [42] were consistent with the results of our study. At the same time, the method of training, the training content, and the method of evaluation of the participants were different from ours. Adherence to the treatment regimen was another dimension that significantly improved after the intervention. The results of a study by Bahiraei et al. [43], consistent with our study, showed the significant Effect of face-to-face education on adherence to the treatment regimen,

In the present study, the improvement of all aspects of self-management led to self-management in diabetes patients, which was consistent with previous research [39–41, 44, 45]. The results of a systematic review by Lean et al. [46] 37 research have shown that frequent care and self-management instruction can improve the prognosis of severe mental illnesses. Good instructional models, like the PRECEDE model, should be used to implement optimal self-management, which is the cornerstone of effective diabetes treatment. Within the framework of the PRECEDE paradigm, self-management theory was employed in this study. Additionally, role-playing, group discussions, homework, and practical skills that centered on accumulating experiences and using scenario-based education approaches were used in the sessions.

Three months following the intervention, mean systolic and diastolic hypertension decreased. Hypertension is a long-term condition of diabetes caused by a lack of understanding, an unhealthy lifestyle, and poor self-management. The PRECEDE paradigm and an appropriate teaching technique changed diabetes patients' lives and improved their self-management skills, reducing their hypertension. Shen et al. [47]. The intervention significantly impacted hypertension, perceived sensitivity, and self-efficacy. The decrease in hypertension was greater in their study than in ours. In our study, using the PRECEDE model resulted in enhanced knowledge, self-efficacy, lifestyle modification, and self-management, which significantly reduced hypertension. The results of Chen et al. systematic.'s review [48]. Although different education techniques have varying effects on patients' hypertension, educational interventions can improve patients' hypertension in general. The findings of an investigation by Ozoemena et al. [49] indicated that health education substantially influences hypertension in the elderly by enhancing their knowledge and self-care skills, which is consistent with our study. Research by Saffari et al. [50] showed that education through SMS could have a similar effect as face-to-face education in lifestyle modification and hypertension control. In our study, web-based methods, including WhatsApp education, were used as a complementary approach.

1. The study population was diabetes patients with hypertension; therefore, it is recommended to exercise caution while generalizing the results to other patients.

2. Due to the physical condition of some patients (decrease in concentration and tolerance), sessions were held with short breaks.

3. Use of a self-report questionnaire to collect data.

4. The participants in the control and intervention groups were in contact with each other outside the sessions, and it was possible to exchange information related to the interventions. At the same time, before the study, the necessary recommendations were given to the participants.

## Conclusion

The educational intervention significantly affected lifestyle modification, improved self-management, and hypertension control in patients with diabetes. They were using effective educational processes in the context of efficient educational theories and techniques by reducing drug consumption and reducing the length of hospital stay of patient's leads to a reduction in treatment and pharmaceutical costs and also a reduction in complications. Therefore, the precede model was found to be a non-invasive, non-pharmacological, cost-effective method without complications and a complementary action along with other methods in treating diabetic patients.

Also, further investigations are recommended be performed on other chronic diseases using different models.

## Data Availability

The datasets used and/or analyzed during the current study are available from the corresponding author upon reasonable request.

## Abbreviation

WHO: World Health Organization

## References

[1] McGill RL, Bragg-Gresham JL, He K, Lacson EK, Miskulin DC, Saran R (2020). Chronic disease burdens of incident US dialysis patients, 1996–2015. Clin Nephrol.

[2] Petrie JR, Guzik TJ, Touyz RM (2018). Diabetes, hypertension, and cardiovascular disease: clinical insights and vascular mechanisms. Can J Cardiol.

[3] Zhang X, Imperatore G, Thomas W, Cheng YJ, Lobelo F, Norris K (2017). Effect of lifestyle interventions on glucose regulation among adults without impaired glucose tolerance or diabetes: a systematic review and meta-analysis. Diabetes Res Clin Pract.

[4] Wang J, Cai C, Padhye N, Orlander P, Zare M (2018). A behavioral lifestyle intervention enhanced with multiple-behavior self-monitoring using mobile and connected tools for underserved individuals with type 2 diabetes and comorbid overweight or obesity: pilot comparative effectiveness trial. JMIR Mhealth Uhealth.

[5] Soheili S, Pirdehghan Y, Hosseini R. Effect of Lifestyle Educational Intervention on Blood Pressure in Diabetic Patients with Hypertension. J Educ Community Health. 2020:0-.

[6] Gallé F, Di Onofrio V, Cirella A, Di Dio M, Miele A, Spinosa T (2017). Improving self-management of type 2 diabetes in overweight and inactive patients through an educational and motivational intervention addressing diet and physical activity: A prospective study in Naples. South Italy Diabetes Therapy.

[7] Chai S, Yao B, Xu L, Wang D, Sun J, Yuan N (2018). The effect of diabetes self-management education on psychological status and blood glucose in newly diagnosed patients with diabetes type 2. Patient Educ Couns.

[8] Chatterjee S, Davies MJ, Heller S, Speight J, Snoek FJ, Khunti K (2018). Diabetes structured self-management education programmes: a narrative review and current innovations. Lancet Diabetes Endocrinol.

[9] Fereidouni Z, SARVESTANI RS, Hariri G, Kuhpaye SA, Amirkhani M, KALYANI MN. Moving Into Action: The Master Key to Patient Education. The Journal of Nursing Research. 2019;27(1):1. 10.1097/jnr.0000000000000280 PMC6369867 30130272

[10] Iquize RCC, Theodoro FCET, Carvalho KA, Oliveira MdA, Barros JdF, Silva ARd. Educational practices in diabetic patient and perspective of health professional: a systematic review. Brazilian Journal of Nephrology. 2017;39(2):196–204. 10.5935/0101-2800.20170034 29069244

[11] Jeihooni AK, Moradi M (2019). The Effect of educational intervention based on PRECEDE Model on promoting skin cancer preventive behaviors in High School students. J Cancer Educ.

[12] Jeihooni AK, Heidari MS, Harsini PA, Azizinia S (2019). Application of PRECEDE model in education of nutrition and physical activities in obesity and overweight female high school students. Obesity Medicine.

[13] Moshki M, Dehnoalian A, Alami A (2017). Effect of precede–proceed model on preventive behaviors for type 2 diabetes mellitus in high-risk individuals. Clin Nurs Res.

[14] Wang Q, Dong L, Jian Z, Tang X (2017). Effectiveness of a PRECEDE-based education intervention on quality of life in elderly patients with chronic heart failure. BMC Cardiovasc Disord.

[15] Aghamolaei T, Hosseini F, Farshidi H, Madani A, Ghanbarnejad A (2015). The impact of an educational intervention based on PRECEDE-PROCEED model on lifestyle of hypertension patients. J Hypertens.

[16] Azar FE, Solhi M, Darabi F, Rohban A, Abolfathi M, Nejhaddadgar N (2018). Effect of educational intervention based on PRECEDE-PROCEED model combined with self-management theory on self-care behaviors in type 2 diabetic patients. Diabetes Metab Syndr.

[17] Barasheh N, Shakerinejad G, Nouhjah S, Haghighizadeh MH (2017). The Effect of educational program based on the precede-proceed model on improving self-care behaviors in a semi-urban population with type 2 diabetes referred to health centers of Bavi. Iran Diabetes & Metabolic Syndrome: Clinical Research & Reviews.

[18] Azar FEF, Solhi M, Azadi NA, Ziapour A, Lebni JY, Chaboksavar F. The Effect of educational intervention based on precede proceed model on life quality in hypertensive patients. 2020.

[19] Ranaei Y, Alhani F, Kazemnejad A, Mehrdad N (2018). The Effect of lifestyle modification through E-learning on self-management of patients with diabetes. J Nurs Educ.

[20] Soheili S, Pirdehghan Y, Hosseini R (2020). Effect of Lifestyle Educational Intervention on Blood Pressure in Diabetic Patients with Hypertension. J Educ Community Health.

[21] Mohammadi Zeidi I, Pakpour Hajiagha A, Mohammadi ZB (2012). Reliability and validity of Persian version of the health-promoting lifestyle profile. Journal of Mazandaran University of Medical Sciences.

[22] Tol A, MR MT, Mahmoodi G, Alhani F, Shojaeezadeh D, Eslami A, et al. Development of a valid and reliable diabetes self-management instrument: An Iranian version. Journal of Diabetes and Metabolic Disorders. 2011;10:18.

[23] Bazpour M, Gheibizadeh M, Malehi AS, Keikhaei B (2019). The Effect of a Training Program Based on the PRECEDE-PROCEED Model on Lifestyle of Adolescents with Beta-Thalassemia: A Randomized Controlled Clinical Trial. International journal of hematology-oncology and stem cell research.

[24] Jeihooni AK, Ghasemi M, Mobaraei AH, Jamshidi H, Harsini PA. The Application of PRECEDE Model on Preventing Osteoporosis in Women. Clinical nursing research. 2019. 10.1177/1054773819865874 31434510

[25] Koç Z, Özdes EK, Topatan S, Çinarli T, Sener A, Danaci E (2019). The Impact of Education About Cervical Cancer and Human Papillomavirus on Women's Healthy Lifestyle Behaviors and Beliefs: Using the PRECEDE Educational Model. Cancer Nurs.

[26] Kaewchin P, Banchonhattakit P, Chamroen P (2019). The Effects of an Integration of PRECEDE-PROCEED Model and Health Literacy in Behavioral Modification for Weight Control among Overweight and Obesity of Adolescents in the Northeast of Thailand. Indian Journal of Public Health Research & Development.

[27] Chaboksavar F, Solhi M, Azadi NA, Azar FEF. Combination of self-management theory with PRECEDE–PROCEED model to promote life quality in patients with hypertension.

[28] Hlaing PH, Sullivan PE, Chaiyawat P (2019). Application of PRECEDE-PROCEED Planning Model in Transforming the Clinical Decision Making Behavior of Physical Therapists in Myanmar. Front Public Health.

[29] Whatnall M, Patterson A, Hutchesson M (2019). A brief web-based nutrition intervention for young adult university students: Development and evaluation protocol using the precede-proceed model. JMIR research protocols.

[30] Taghdisi M, Borhani M, Solhi M, Afkari M, Hosseini F (2012). The Effect of an education program utilising PRECEDE model on the Quality of Life in patients with type 2 diabetes. Health Educ J.

[31] Jeihooni AK, Ghasemi M, Mobaraei AH, Jamshidi H, Harsini PA (2019). The Application of PRECEDE Model on Preventing Osteoporosis in Women. Clin Nurs Res.

[32] Doshmangir P, Shirzadi S, Tagdisi MH, Doshmangir L (2014). Effect of an educational intervention according to the PRECEDE model to promote elderly quality of life. J Educ Community Health.

[33] Oshvandi K, Salavati M, Ahmadi F, Soltanian A (2018). The Effect of Training on Hemodialysis Patients’ Lifestyle Promotion. Avicenna Journal of Nursing and Midwifery Care.

[34] Shayesteh H, Mansoriyan M, Mirzaie A, Sayehmiri K. Survey of the Effect of educational intervention on the nutrition physical activity and stress management of patients with hypertension among the rural population of Aligoudarz County of Lorestan Province in 2015. scientific journal of ilam university of medical sciences. 2016;24(2):54–62.

[35] Khavoshi N, Tol A, Shojaeizade D, Shamshiri A (2015). Effect of educational intervention on the lifestyle of elderly people referred to clinical centers of Eslamshahr, Iran: application of health belief model. J Nurs Educ.

[36] Chafjiri RT, Shirinkam F, Karimi H (2018). Investigating the Effect of education on health-promoting lifestyle among the elderly of Ramsar in 2017. Journal of family medicine and primary care.

[37] Safabakhsh L, Nazemzadeh M (2013). The Effect of health promotion education on high school students’ lifestyle. Iranian Journal of Medical Education.

[38] Ebrahimi F, Aghamolaei T, Abedini S, Rafati S (2017). Effect of educational intervention using mobile on life style of women who referred to health centers in Bandar Abbas. Iranian Journal of Health Education and Health Promotion.

[39] Habibzadeh H, Sofiani A, Alilu L, Gillespie M (2017). The Effect of group discussion-based education on self-management of adults with type 2 diabetes mellitus compared with usual care: A randomized control trial. Oman Med J.

[40] Weng Y, Zhang D, Lin Q, Chen Q, Minghua H (2018). Effect of omni-directional health education on self management level and blood glucose control in type 2 diabetic patients. Chinese Journal of Integrated Traditional and Western Medicine in Intensive and Critical Care.

[41] Liu H, Zhou X, Song M, Bai Y, Xiaohong L (2018). Effect of motivational interviewing health education on self-management behavior and lung function of elderly patients with stable chronic obstructive pulmonary disease in the community. Chinese Journal of Geriatrics.

[42] Mayor S (2015). Simple education reduces inappropriate blood glucose self monitoring in type 2 diabetes, study shows. BMJ.

[43] Bahiraei N, Dehghani M, Khachian A (2019). The Effect of educational program on self-management of patients with epilepsy: a Randomized Clinical Trial Study. Avicenna Journal of Nursing and Midwifery Care.

[44] Ahmed HAM, Aljaber NY, Ahmed EAA, Ali LMG. The Effect of Developing and Implementing Health Education and Nutrition Training Program on Self-Management Practices among Patients with Iron Deficiency Anemia, Alexandria Main University Hospital, Egypt. International Journal of Innovative Research in Medical Science. 2018;3(05):2013 to 21- to 21.

[45] Ren Q, Lian M, Liu Y, Thomas-Hawkins C, Zhu L, Shen Q (2019). Effects of a transtheoretical model-based WeChat health education programme on self-management among haemodialysis patients: A longitudinal experimental intervention study. J Adv Nurs.

[46] Lean M, Fornells-Ambrojo M, Milton A, Lloyd-Evans B, Harrison-Stewart B, Yesufu-Udechuku A (2019). Self-management interventions for people with severe mental illness: systematic review and meta-analysis. Br J Psychiatry.

[47] Shen Y, Wang T, Gao M, Hu K, Zhu X, Zhang X (2020). Effectiveness evaluation of health belief model-based health education intervention for patients with hypertension in community settings. Zhonghua yu Fang yi xue za zhi [Chinese Journal of Preventive Medicine].

[48] Chen Y, Li X, Jing G, Pan B, Ge L, Bing Z (2020). Health education interventions for older adults with hypertension: A systematic review and meta-analysis. Public Health Nurs.

[49] Ozoemena EL, Iweama CN, Agbaje OS, Umoke PC, Ene OC, Ofili PC (2019). Effects of a health education intervention on hypertension-related knowledge, prevention and self-care practices in Nigerian retirees: a quasi-experimental study. Archives of Public Health.

[50] Saffari M, Sanaeinasab H, RASHIDI JH, SEPANDI M, SAMADI M, AZAD ME. A Comparison Between Impact of a Health Education Program Using In-situ Training and Text-Messaging on Lifestyle and Blood Pressure in Military Personnel at Risk of Hypertension. JOURNAL OF HEALTH EDUCATION AND HEALTH PROMOTION. 2019;7(1):74–83.

